# Predictive Indicators of Brain Metastasis-Free Survival in Patients With Lung Cancer at a Chinese Cancer Center

**DOI:** 10.7759/cureus.19995

**Published:** 2021-11-29

**Authors:** Hui Ye

**Affiliations:** 1 Radiology, Sun Yat-sen University Cancer Center, Guangzhou, CHN

**Keywords:** leukoaraiosis, brain metastasis, squamous cell lung carcinoma, lung cancer, survival analyses

## Abstract

Introduction

Metastasis tumors of the brain derive mostly from lung cancer, breast cancer, melanoma, and more commonly among lung cancer patients. Once brain metastasis is diagnosed, the prognosis of untreated patients is shown to be very poor. In this study, we describe the clinical and pathological features of patients with lung cancer at our institution from 2009 to 2021. We also examined factors like gender, type, size, and location of the primary tumor and leukoaraiosis level at the first visit are associated with patients’ brain metastasis-free survival (the time free of brain metastases since the first diagnosis of lung cancer).

Materials and methods

We retrospectively reviewed patients with a final histologic diagnosis of lung cancer from September 2009 to January 2015. The evaluation included history, physical examination, and contrast-enhanced computerized tomography of the chest. Contrast-enhanced magnetic resonance imaging of the head was performed at the first visit and following treatment. The patients’ age, gender, tumor size, histology type, location of the lung tumor, and leukoaraiosis level at the first visit were recorded and correlated to the patients’ brain-metastasis-free survival time.

Results

The study included 68 patients - 39 males and 29 females -with a mean age of 55.15 years (range 35-69 years). Adenocarcinoma was diagnosed in 47 patients (22 males and 25 females), Squamous carcinoma was diagnosed in 12 patients (9 males and 3 females), non-small cell lung carcinoma was diagnosed in 3 patients (2 males and one female), one male patient had the diagnosis of adenosquamous tumor and 1 male patient had the diagnosis of neuroendocrine tumor. Tumor size was <3 cm in 19 patients, 3-5 cm in 29 patients, 5-10 cm in 17 patients, and three other patients’ size was hard to measure. Of the 68 patients, 10 patients were detected as brain metastasis by magnetic resonance imaging at first diagnosis, 22 patients were diagnosed as brain metastasis during the follow-up visits, 36 patients were not found as brain metastasis until the last visit. According to the Cox regression univariate analysis, two factors were correlated to shorter brain metastasis-free survival: Not-squamous lung carcinoma (SCC) and higher location of the tumor. The multivariate statistical analysis correlated two factors to shorter brain metastasis-free survival: non-SCC histology type and age≥62.

Conclusions

In conclusion, we found that SCC had a lower incidence of brain metastasis in patients than other lung cancer types. According to the Cox regression multivariate analysis, age <62 and SCC were two protective factors of brain metastasis. According to the Cox regression univariate and analysis. The lower location of the tumor was the protective factor of brain metastasis. According to the Cox regression univariate analysis, other parameters, such as gender or tumor size, did not have a role in brain metastasis-free survival in these patients.

## Introduction

According to a new report from GLOBOCAN, lung cancer remained the leading cause of cancer death (18% of the total cancer deaths) [1]. Brain metastases (BM) remain an important cause of morbidity and mortality in patients with lung cancer [2]. Silent BM has been detected more since the advancements in medical images and treatment of primary cancers and heightened physician and patient awareness of BM [3-5]. A majority of BM occurs at the gray-white junction due to the hematogenous spread of tumor emboli, which become entrapped in the small branches of terminal arteries found at this junction [6]. Local treatment such as radiation or surgery has been applied to control metastatic brain lesions [7].

Articles showed that Squamous cell carcinoma (SCC) was related to a lower risk of BM compared with other types of lung cancer, which was consistent with the previous results. The impact of gender T-stage and N-stage and age was widely discussed in many studies. Tumor size and metastatic site were mentioned in some study too. Leukoaraiosis (LA), also known as white matter hyperintensities (WMH), is usually found in computed tomography (CT) and magnetic resonance imaging (MRI) brain scans’ images of elderly individuals [8,9]. LA was thought to play a role in stroke, dementia, and cognitive impairment [10]. It was reported that small vessel ischemic disease plays a protective role against the development of BM in lung cancer patients [11]. According to a previews study, factors predictive of brain progression were age < or =62 years (RR: 2.5, 95% CI: 1.33-4.76 and P = 0.004), T4 tumor status (RR: 3.75, 95% CI: 1.72-8.21 and P = 0.0009), N2-3 (RR: 2.61, 95% CI: 1.32-5.15 and P = 0.0057), and adenocarcinoma (RR: 3.39, 95% CI: 1.78-6.46 and P = 0.0002) [12]. Another study found that factors predictive of brain progression were age ≤62 years, T4 tumor status, and adenocarcinoma, and no aspect of treatment plays a role in the frequency of this type of metastasis [13]. Gender, LA level, tumor size, and metastatic site were mentioned in some study too.

In this study, we describe the clinical and pathological features of patients with lung cancer at our institution from 2009 to 2021. We also examined if these features, including age, gender, type, size, and location of the primary tumor and LA level at the first visit, are associated with patients’ brain metastasis-free survival (the time free of brain metastases since the first diagnosis of lung cancer).

## Materials and methods

We retrospectively reviewed patients with a final histologic diagnosis of lung cancer from September 2009 to January 2015. The evaluation included history, physical examination, contrast-enhanced computerized tomography of the chest, and contrast-enhanced magnetic resonance imaging of the head. All patients underwent pulmonary tumor puncture biopsy or surgery for pathological analysis performed in the clinical pathology laboratory (Department of Pathology, Sun Yat-sen University Cancer Center). Tumors were classified into adenocarcinoma (ADC), SCC, adenosquamous lung carcinoma (ASLC), small cell lung carcinoma (SCLC), and neuroendocrine. No patient received prophylactic cranial irradiation.

Chest CT scans were performed as a part of the diagnostic work-up using one of the four helical scanners: Siemens Somatom Force, Philips Brilliance ICT, GE Discovery CT750 HD, or Toshiba Aquilion TSX-101A. The CT scans were obtained from the supraclavicular regions to the upper abdomen following intravenous administration of a contrast agent，using 5mm thick sections. MRI images are the most common way to detect brain metastasis, contributing higher frequency of the diagnosis of BMs [14,15]. In this study, MRI scanning was performed using a 3 T unit: Siemens Magneton Trio A Tim, GE Discovery MR750w, GE Discovery MR750, or Philips Achieve 781-278 3.0T. T1-weighted images. T2-weighted and T2 flair images were obtained in the axial plane with 5mm sections and 1 mm intersection gap. Axial (5mm sections and 1 mm intersection gap), coronal (5mm sections and 1 mm intersection gap), and thin layer sagittal (1mm sections and 0 mm intersection gap) T1-weighted images were obtained for contrast MR studies after intravenous administration of gadolinium contrast agent (0.1 mmol/kg). One radiologist measured tumor size (with more than five years of experience) based on chest CT images.

LA is defined as a high signal on Flair and T2 sequences and is usually hard to find on T1 sequences or is mildly low signal on T1 sequences. Using the Fazekas’ score, we graded cerebral white matter sparseness for ventricular periventricular white matter hyperintensities (PWMH) and deep white matter hyperintensities (DWMH). PWMH was graded as 0=absence,1=’caps’ or pencil-thin lining, 2= smooth’ halo’, 3= irregular PVH extending into the DWM. Separated DWMH signals were rated as 0=absence, 1=punctate foci, 2=beginning confluence of foci,3=large confluence areas [16]. The Fazekas’ score was calculated separately and added up a total PWMH and DWMH range from zero (have no signal of LA) to six.

BMs were defined as focal intra-axial lesions with T1 signal intensity enhancement after gadolinium intravenous administration and a variable degree of perilesional edema that was hyperintense on FLAIR and T2 weighted images and slightly hypointense on T1 weighted images. Brain metastases were diagnosed based on brain MRI scans interpreted by one radiologist with more than five years of experience.

Survival time was measured in units of days from the first visit when the cancer was first detected. The patients’ follow-up ended in October 2021, with a mean time of observation of two years (864 days). We performed brain enhanced MRI every 4-6 months for the first years and then every 6-12 months during the follow-up. Once diagnosed, patients will receive various treatments such as surgery, chemotherapy, radiation, and targeted therapies.

The patients’ age, gender, tumor size at the first visit, pathological type, and LA level at the first visit were recorded and correlated to the patients’ BM-free survival time. The Statistical Package for Social Sciences software (SPSS for Windows, version 26.0, SPSS Inc) was used for statistical analysis. Kaplan-Meier statistics analysis table and survival curves used the log-rank test and Breslow (Generalized Wilcoxon). Univariate and multivariate analysis for the prognostic factors uses the Cox hazard-regression model, including relative risk(RR), probability, and 95% confidence interval. The level of statistical significance was 5%.

## Results

The study included 68 patients - 39 males and 29 females -with a mean age of 55.15 years (range 35-69 years). ADC was the diagnosis in 47 patients (22 males and 25 females), SCC was the diagnosis in 12 patients (9 males and 3 females), SCLC was the diagnosis in 4 male patients, non-SCLC (no further pathology was reported)was the diagnosis in 3 patients (2 males and 1 female), one male patient had the diagnosis of adenosquamous lung carcinoma and one male patient had the diagnosis of neuroendocrine tumor. Tumor size was <3 cm in 19 patients, 3-5 cm in 29 patients, 5-10 cm in 17 patients, and three other tumor size was hard to measure.LA level was classified into four levels: 23 patients at level zero, 28 patients at level one, 16 patients at level two, and one at level three. Of the 68 patients, 10 patients were detected as BM by magnetic resonance imaging at first diagnosis, 22 patients were diagnosed as BM during the follow-up visits, 36 patients were not found as BM until the last visit.

Kaplan Meier survival analysis by Log Rank test and Breslow test was shown in table 1. SCC patients had a lower probability of having BM than the other lung cancer types with the Kaplan Meier survival analysis by Log Rank test (P=0.013) and Breslow test (P=0.026). Tumors located at the inferior lobe had a lower probability of having BM than the tumor located at other lung areas with the Kaplan Meier survival analysis by Breslow test(P=0.040). Other factors, including LA level at the first visit, gender, age<62 or not (age group in table 1), size <3cm or not (size group in table 1), and ADC or not, had low confidence of their influence of the BM’s happening.

**Table 1:** Kaplan Meier survival analysis by Log Rank test and Breslow test. LA level’s degrees of freedom of Kaplan Meier survival analysis is 3, other five factors’ degrees of freedom of Kaplan Meier survival analysis are 1. BM- brain metastasis; LA- leukoaraiosis; SCC- squamous carcinoma; ADC- adenocarcinoma; Sig- significance

	LA level		SCC or not		Gender		ADC or not		Size group		Site group		Age group	
	Chi-Square	Sig.	Chi-Square	Sig.	Chi-Square	Sig.	Chi-Square	Sig.	Chi-Square	Sig.	Chi-Square	Sig.	Chi-Square	Sig.
Log Rank (Mantel-Cox)	6.190	0.103	6.113	0.013	0.347	0.556	2.576	0.108	0.062	0.803	3.744	0.053	3.348	0.067
Breslow (Generalized Wilcoxon)	4.832	0.185	4.933	0.026	0.116	0.734	1.537	0.215	0.740	0.390	4.218	0.040	3.079	0.079

**Figure 1:**
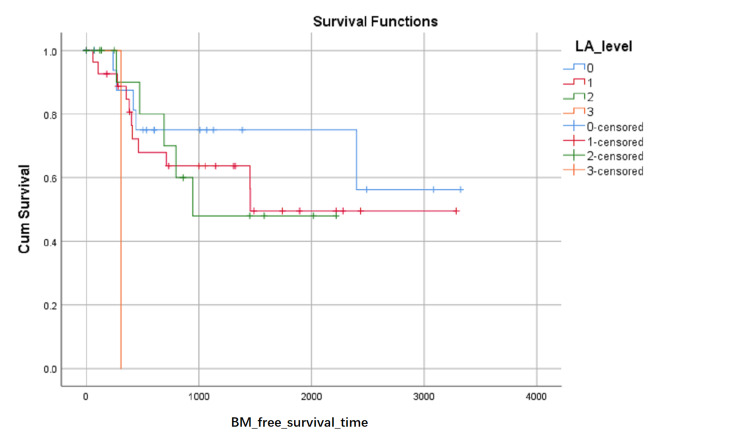
BM-free survival rates based on LA level at the first visit. The x-axis indicates days from the first visit and the y-axis indicates the proportion of BM-free surviving patients. BM: brain metastasis LA: leukoaraiosis

**Figure 2:**
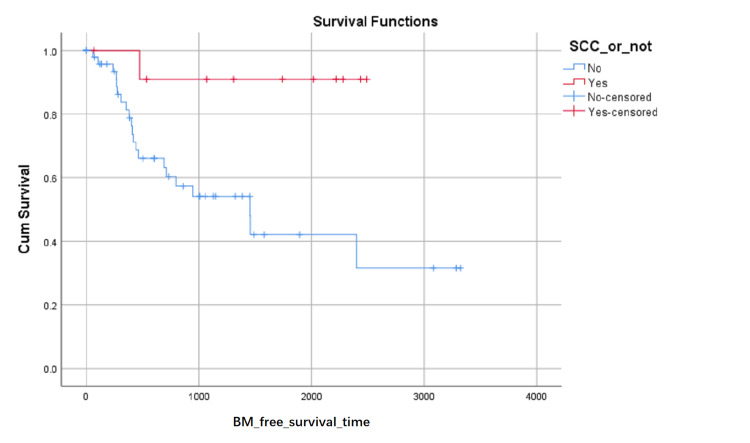
BM-free survival rates based on SCC or not. The x-axis indicates days from the first visit and the y-axis indicates the proportion of BM-free surviving patients. BM: brain metastasis. SCC: squamous carcinoma.

**Figure 3:**
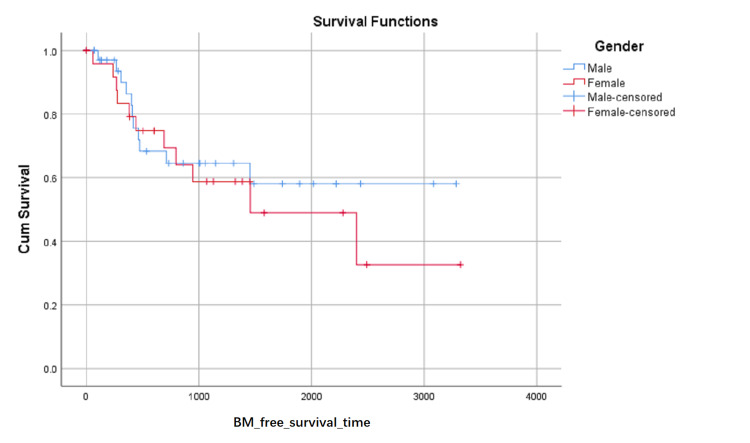
BM-free survival rates based on Gender. The x-axis indicates days from the first visit and the y-axis indicates the proportion of BM-free surviving patients. BM: brain metastasis

**Figure 4:**
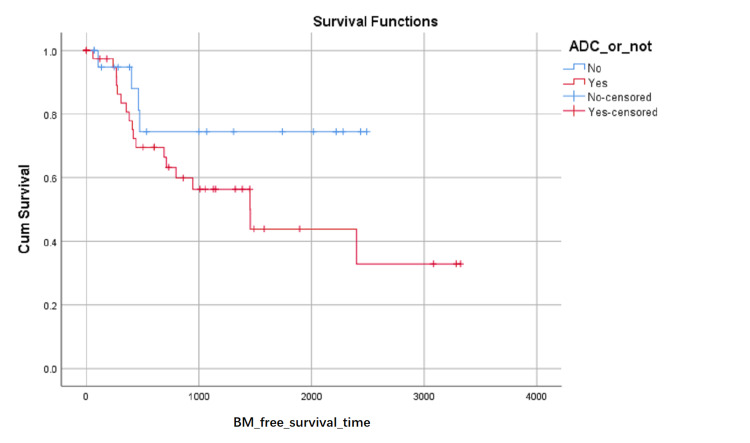
BM-free survival rates based on ADC or not. The x-axis indicates days from the first visit and the y-axis indicates the proportion of non-BM surviving patients. BM: brain metastasis. ADC: adenocarcinoma.

**Figure 5:**
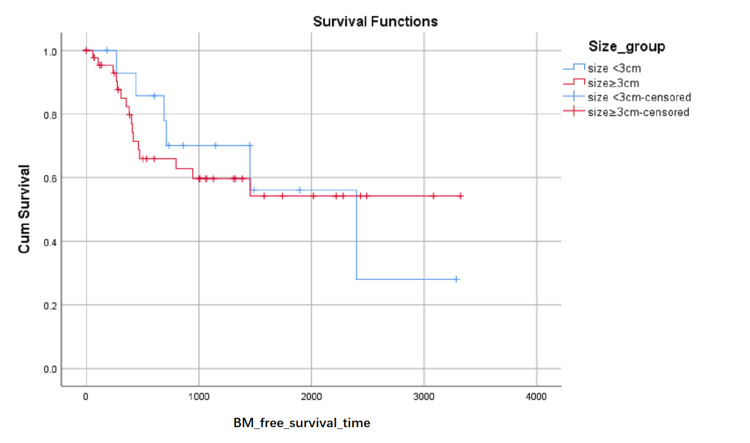
BM-free survival rates based on size smaller than 3cm or not. The x-axis indicates days from the first visit and the y-axis indicates the proportion of BM-free surviving patients. BM: brain metastasis.

**Figure 6:**
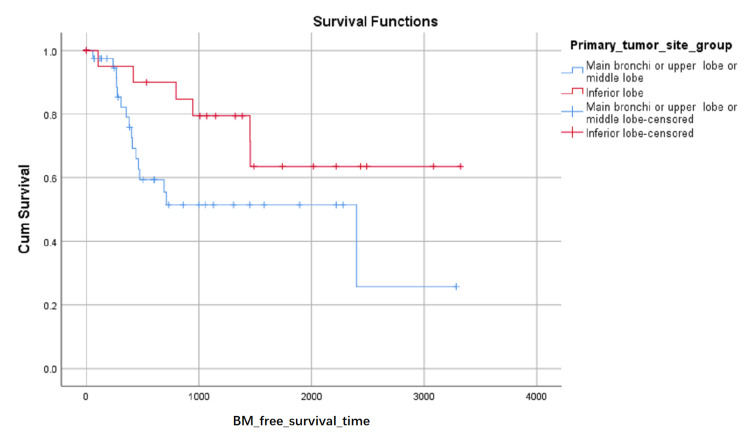
BM-free survival rates based on primary tumor site group by inferior lobe or other areas of the lung. The x-axis indicates days from the first visit and the y-axis indicates the proportion of BM-free surviving patients. BM: brain metastasis

**Figure 7:**
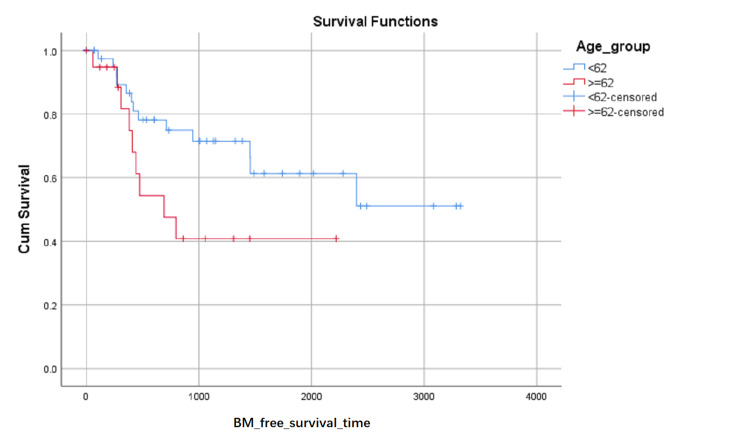
BM-free survival rates based on age younger than 62 or not. The x-axis indicates days from the first visit and the y-axis indicates the proportion of BM-free surviving patients. BM: brain metastasis.

**Figure 8:**
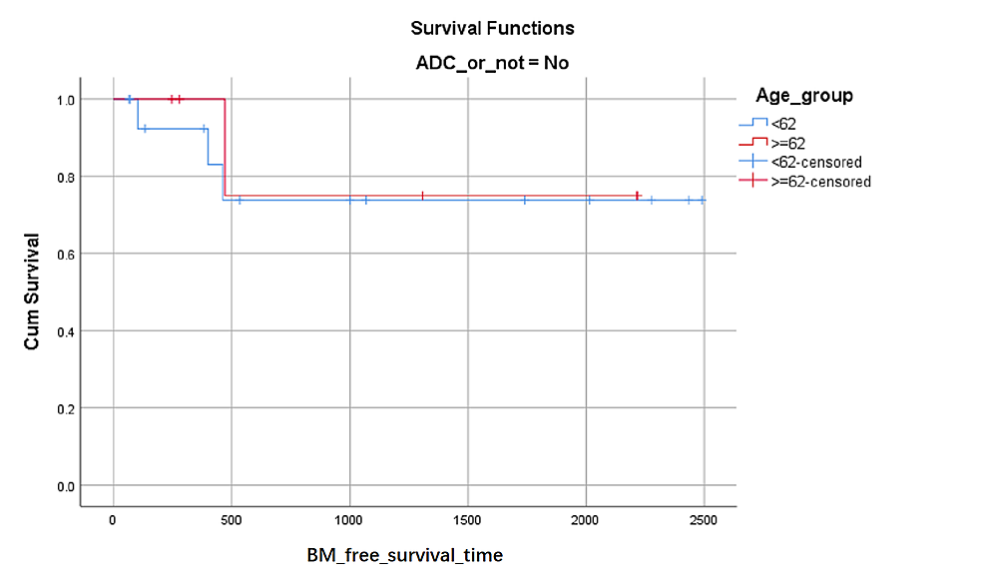
BM-free survival rates based on age older than 62 or not among non- ADC patients. The x-axis indicates days from the first visit and the y-axis indicates the proportion of BM-free surviving patients. BM: brain metastasis. ADC: adenocarcinoma

**Figure 9:**
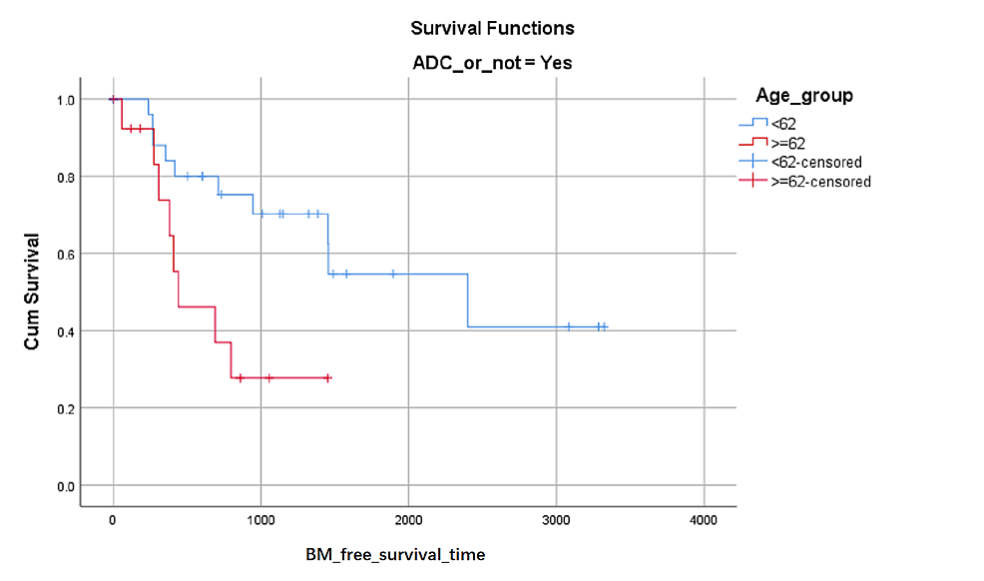
BM-free survival rates based on age older than 62 or not among ADC patients. The x-axis indicates days from the first visit and the y-axis indicates the proportion of BM-free surviving patients. BM: brain metastasis. ADC: adenocarcinoma

BM-free survival rates based on LA level at the first visit were shown in (Figure 1). Although the influence on BM-free survival of the LA level had not statistically significant (0.103 with Log Rank test), it can be seen in (Figure 1) that the proportion of BM-free surviving patients reduced in turn of the zero level, first level, and second level after about 1000 days later from the first visit, but the proportion of BM-free surviving patients reduced in turn of the second level, zero level and first level between about 348 and about 683 days from the first visit. BM-free survival rates based on SCC or not were shown in (Figure 2) and indicated that histology type of SCC had higher BM-free survival rates than other types. BM-free survival rates based on gender were shown in (Figure 3), ADC or not was shown in (Figure 4), based on size <3cm or not was shown in (Figure 5), and BM-free survival rates based on tumor located at inferior lobe or not was shown in (Figure 6). The factors are shown in Figures 3-6 have no statistical significance on the BM-free survival time. BM-free survival rates based on age<62 or not (Figure 7). Since curves are crossed over, the test may not be appropriate, and alternative methods should be considered. An intensive study found that when histology type was ADC, the patients older than 62 had lower BM-free survival rates than patients younger than 62. Kaplan Meier survival analysis of patients’ first visit age <62 or not, using Log Rank test and Breslow test and stratified by whether they are ADC or not, was shown in Table 2. Survival functions of the age group, while the histology type was or was not ADC, were shown in Figures 8, 9.

**Table 2:** Kaplan Meier survival stratification analysis of age group. BM: brain metastasis. ADC:adenocarcinoma. Sig.:significance

Pairwise Comparisons						
	ADC_or_not	Age_group	<62		>=62	
			Chi-Square	Sig.	Chi-Square	Sig.
Log Rank (Mantel-Cox)	No	<62			0.069	0.793
		>=62	0.069	0.793		
	Yes	<62			6.238	0.013
		>=62	6.238	0.013		
Breslow (Generalized Wilcoxon)	No	<62			0.194	0.660
		>=62	0.194	0.660		
	Yes	<62			5.227	0.022
		>=62	5.227	0.022		

Multivariate Cox regression analysis factors were selected according to Kaplan Meier survival stratification analysis and Cox regression univariate analysis. According to the Cox regression univariate analysis, one factor was correlated to higher BM-free survival time: SCC histology type (95.0% CI from 0.016 to 0.892) (Table 3). The multivariate statistical analysis correlated two factors to higher BM-free survival rates: SCC histology type and age<62 (Table 4). The result indicates SCC patients have a lower probability of BM among lung cancer patients. In comparison, the histology type of the lung cancer was ADC, age<62 or not played an essential role in predicting the occurrence of BM.

**Table 3:** Univariate Cox regression analysis. SE: square deviation. Exp(B): the relative risk between the groups. LA: leukoaraiosis. SCC: squamous carcinoma. ADC: adenocarcinoma. Sig.: significance.

Variables in the Equation								
	B	SE	Wald	df	Sig.	Exp(B)	95.0% CI for Exp(B)	
							Lower	Upper
LA_level			4.461	3	0.216			
level 1 vs level 0	0.407	0.546	0.555	1	0.456	1.502	0.515	4.380
Level 2 vs level 0	0.456	0.644	0.501	1	0.479	1.578	0.446	5.577
Level 3 vs level 0	2.434	1.154	4.449	1	0.035	11.406	1.188	109.513
Gender	0.251	0.427	0.345	1	0.557	1.285	0.557	2.967
Age_group	0.795	0.446	3.184	1	0.074	2.216	0.925	5.308
Primary_tumor_site_group	-0.911	0.486	3.517	1	0.061	0.402	0.155	1.042
Size≥3cm vs Size<3cm	0.120	0.479	0.062	1	0.803	1.127	0.440	2.885
ADC or not	0.863	0.554	2.424	1	0.119	2.369	0.800	7.018
SCC_or_not	-2.126	1.027	4.288	1	0.038	0.119	0.016	0.892

**Table 4:** Multivariate Cox regression analysis SE: square deviation. Exp (B): the relative risk between the groups. LA: leukoaraiosis. SCC: squamous carcinoma. ADC: adenocarcinoma

Variables in the Equation								
	B	SE	Wald	df	Sig.	Exp(B)	95.0% CI for Exp(B)	
							Lower	Upper
Age_group	0.997	0.499	3.986	1	0.046	2.709	1.018	7.206
Primary_tumor_site_group	-0.612	0.559	1.197	1	0.274	0.542	0.181	1.623
SCC_or_not	-2.867	1.242	5.331	1	0.021	0.057	0.005	0.648
ADC_or_not	-0.354	0.714	0.246	1	0.620	0.702	0.173	2.842
Size_group	0.804	0.533	2.270	1	0.132	2.234	0.785	6.355

## Discussion

With the multivariate analysis conducted by A Bajard, factors predictive of BM in localised NSCLC were age ≤62 years (RR: 2.5, 95% CI: 1.33-4.76 and P = 0.004), ADC (RR: 3.39, 95% CI: 1.78-6.46 and P = 0.0002) T4 tumor status (RR: 3.75, 95% CI: 1.72-8.21 and P = 0.0009) and N2-3 (RR: 2.61, 95% CI: 1.32-5.15 and P = 0.0057) [13]. However, in the present study, when histology type was ADC, the patients older than 62 had lower BM-free survival rates than patients younger than 62 (RR: 2.709, 95% CI: 1. 1.018-7.206 and P = 0.046). The study by A Bajard time was 1977-2001, and the object of the present study was 2009-2021. Times’ differences may cause the improvement of treatments and different results of research. This difference may also be caused by the small population scale and the need to have further exploration.

The study of Harvey J et al. showed more patients with BM among patients with non-SCC than SCC histology (44% v 32%, respectively, at three years; P = 0.037) with univariate analysis [17]. In a study by Chen Allen M et al., the 5-year estimates of brain metastasis-free survival for patients with SCC and non-SCC were 57% and 34%, respectively (P = 0.02) [18]. The research of this article showed that SCC was related to a lower risk of BM compared with other types of lung cancer, which was consistent with the previous results. Shi Ann A, in their study, showed that there was no significant difference in tumor histology, staging, or distribution between symptomatic or asymptomatic patients with NSCLC with BM, and the odds of BM were more significant in those with ADC or large-cell carcinoma [19]. Multivariate analysis conducted by A Bajard also considered ADC pathological type as a factor of BM in localized NSCLC (RR: 3.39, 95% CI: 1.78-6.46 and P = 0.0002) [13]. In the current study, ADC had no effect on the BM-free survival time with a statistical significance of 0.05. The significance of the Log Rank test was 0.108. Thus further study may be promising to determine whether the ADC type would be a dangerous factor of BM.

There was no significant difference in brain metastasis-free survival according to gender, age, initial T-stage, or neoadjuvant modality (P >0.05, for all), mentioned in the study by Chen Allen M et al. [18]. It was reported that women (43% vs. 35%) and younger patients had more metastases to the nervous system [20]. According to the Metropolitan Detroit Surveillance analysis, the incidence of BM in patients with nonmetastatic lung cancer varies according to histology, age, and sex. BM is associated with worse survival for patients with NSCLC but not those with SCLC [2]. Gender was not a significant influence factor in the current study according to Kaplan Meier survival analysis and the Cox regression univariate analysis.

According to previous studies by Mazzone PJ et al., vascular changes in the brain are protective against the development of brain metastases in lung cancer patients [9]. In the study by Carlo Cosimo Quattrocchi et al., volumes of BM at the first MR diagnosis in a sample of advanced cancer patients and the group of lung cancer patients were significantly lower if brain white matter T2 hyperintensities were present and suggested that WMH may represent a clinical MRI biomarker of brain micro-environment resistance to the occurrence of brain metastases [11]. The present result suggested that the LA level at the first visit appears to be meaningless as a factor of BM. This difference may be caused by the lack of patients with high-level LA(≥3) at the first visit.

In a recent study, there was statistical significance between tumor size and metastatic site in patients with stage IV NSCLC and for brain or lung metastasis; The larger the tumor, the higher the risk of brain or lung metastasis [21]. An analysis of 975 patients with early-stage NSCLC revealed that younger age (hazard ratio [HR], 1.03 per year), larger tumor size (HR, 1.26 per cm), lymphovascular space invasion (HR, 1.87), and hilar lymph node involvement (HR, 1.18) were associated with an increased risk of developing brain metastases On multivariate analysis, [22]. In this study, the tumor located at the inferior lobe was a dangerous factor according to the Kaplan Meier survival analysis, but not significantly with Cox regression univariate analysis. The relation between BM’s happening and the size of the original tumor size <3cm or not was not statistically significant and still needs further studies.

The study has some limitations. First, only a limited number (22) of no-censor data was presented. Cox regression can process the deleted data with different survival time distributions, but the results will be biased because of the deletion. Therefore, better results need a more extensive study. Secondly, initial N-stage or T-stage were not analyzed in the current study. Higher T-stage and N-stage contribute to the occurrence of BM, mentioned in the study by A Bajard et al. [13], a study by Chen Allen M et al. [20], and a study by Hubbs JL et al. [22].

Last but not least, the specific treatment was not collected in this study. Although a previous study found that no aspect of treatment plays a role in the frequency of NSCLC [13], the early treatment may help inhibit brain metastases. For example, neoadjuvant modality was mentioned as a significant factor in the study by Chen Allen M et al. [20], and one retrospective data suggested a potential role for gefitinib and erlotinib in advanced NSCLC patients with brain metastases which have failed to respond to radiotherapy and patients with EGFR mutations [23].

## Conclusions

We found that the incidence of BM in patients with lung cancer is related to histological type (SCC was protection factor), age≥62, and location of the lung cancer. According to the Cox regression univariate analysis, other parameters, such as gender, tumor size, LA level at the first visit, did not have a role in BM-free survival in these patients. The study needs larger data to find out the reasons for the difference of the factors to BM with previous studies.
